# Systematic review of predictive performance of injury severity scoring tools

**DOI:** 10.1186/1757-7241-20-63

**Published:** 2012-09-10

**Authors:** Hideo Tohira, Ian Jacobs, David Mountain, Nick Gibson, Allen Yeo

**Affiliations:** 1 School of Primary, Aboriginal and Rural Health Care, The University of Western Australia, M516 The University of Western Australia, 35 Stirling Highway, Crawley, WA, 6009, Australia; 2 Department of Surgery, Sir Charles Gairdner Hospital, Hospital Avenue, Nedlands, WA, 6009, Australia

**Keywords:** ISS, NISS, TRISS, ICISS, AUROC, Outcome prediction

## Abstract

Many injury severity scoring tools have been developed over the past few decades. These tools include the Injury Severity Score (ISS), New ISS (NISS), Trauma and Injury Severity Score (TRISS) and International Classification of Diseases (ICD)-based Injury Severity Score (ICISS). Although many studies have endeavored to determine the ability of these tools to predict the mortality of injured patients, their results have been inconsistent. We conducted a systematic review to summarize the predictive performances of these tools and explore the heterogeneity among studies. We defined a relevant article as any research article that reported the area under the Receiver Operating Characteristic curve as a measure of predictive performance. We conducted an online search using MEDLINE and Embase. We evaluated the quality of each relevant article using a quality assessment questionnaire consisting of 10 questions. The total number of positive answers was reported as the quality score of the study. Meta-analysis was not performed due to the heterogeneity among studies. We identified 64 relevant articles with 157 AUROCs of the tools. The median number of positive answers to the questionnaire was 5, ranging from 2 to 8. Less than half of the relevant studies reported the version of the Abbreviated Injury Scale (AIS) and/or ICD (37.5%). The heterogeneity among the studies could be observed in a broad distribution of crude mortality rates of study data, ranging from 1% to 38%. The NISS was mostly reported to perform better than the ISS when predicting the mortality of blunt trauma patients. The relative performance of the ICSS against the AIS-based tools was inconclusive because of the scarcity of studies. The performance of the ICISS appeared to be unstable because the performance could be altered by the type of formula and survival risk ratios used. In conclusion, high-quality studies were limited. The NISS might perform better in the mortality prediction of blunt injuries than the ISS. Additional studies are required to standardize the derivation of the ICISS and determine the relative performance of the ICISS against the AIS-based tools.

## Background

Many scoring systems to assess injury severity have been developed over the past few decades. The need to improve the quality of trauma care has led researchers to develop more accurate tools that allow physicians to predict the outcomes of injured patients. The Abbreviated Injury Scale (AIS) was the first comprehensive injury severity scoring system to describe injuries and to measure injury severity [1]. Because the AIS cannot measure the overall injury severity of a patient with multiple injuries, tools that can measure the overall severity of multiple injuries were developed using the AIS. These tools include the Injury Severity Score (ISS) [2], the New Injury Severity Score (NISS) [3] and the Trauma and Injury Severity Score (TRISS) [4].

AIS codes are not always available for all injured patients because of the limited resources available for maintaining a trauma registry. However, the International Classification of Diseases (ICD) codes are routinely collected in administrative databases, including morbidity or mortality databases. Osler et al. introduced the ICD-based Injury Severity Score (ICISS) to overcome the unavailability of AIS-based tools [5]. It was reported that the ICISS performed as well as the AIS-based tools in predicting trauma patient outcomes [6-17].

Many researchers have studied the predictive performances of injury severity scoring tools. Their results, however, were inconsistent. This disparity may be due to the differences in study populations and the differences between the formulas used to calculate severity. For example, the ICISS employs at least two types of formulas for its calculation: a product of multiple survival risk ratios (SRRs), referred to as the traditional ICISS, and the use of a single worst injury (SWI) risk ratio. In 2003, Kilgo et al. first reported that the SWI performed better than the traditional ICISS [18]. The superiority of the SWI remains uncertain because of conflicting results from another researcher [19].

The predictive performances of the TRISS and ICISS have rarely been compared. The TRISS utilizes a logistic regression model that incorporates the ISS as a predictor; therefore, the TRISS intuitively outperforms the ISS. In contrast to the TRISS, the ICISS is based on a multiplicative model that uses SRRs. Because the TRISS and ICISS are based on different mathematical models, the superiority of one tool over the other remains inconclusive.

Although several traditional narrative reviews have been conducted, these reviews typically focused on how each tool was established and how each score was derived [20-24]. A few reviews have addressed the predictive performances of injury severity scoring tools [21,23]. However, the methodologies used for selecting studies were unclear, and the interpretation of the results was subjective. It is more appropriate to review studies in a systematic manner to best integrate all of the evidence. Currently, there is no systematic review that evaluates the predictive performances of injury severity scoring tools.

### Aims

In this systematic review, we aimed to summarize the ability of the injury severity scoring tools that are currently in use to predict the mortality of injured patients. We also aimed to explore the potential sources of the heterogeneity among studies to better understand comparative studies of injury severity scoring tools.

## Methods

### Injury severity scoring tools

To investigate predictive performances, we chose the following injury severity scoring tools: the ISS [2], the NISS [3], the TRISS [4] and the ICISS [25]. These tools are hereafter referred to as the “target tools.” These target tools were selected because they were frequently found in injury research articles. We included the TRISS in the target tools to determine the superiority of the TRISS over the ICISS or vice versa.

We subdivided the TRISS and the ICISS further when reporting their predictive performance. We classified the TRISS into two types: the TRISS that used coefficients derived from the Major Trauma Outcome Study (MTOS TRISS) and one that used coefficients derived from non-MTOS populations (non-MTOS TRISS).

We categorized the ICISS into four subgroups based on the type of formula and SRR. There are two types of formula, as described previously. There also are two types of SRR: traditional SRR and independent SRR. An SRR is calculated by dividing the number of survivors with a given ICD code by the total number of patients with the ICD code. Traditional SRRs are calculated using not only single-trauma patients but also multiple-trauma patients, whereas independent SRRs are derived using cases with a single injury only. The independent SRRs are mathematically correct because the traditional SRRs violate the independence assumption of probability. Based on the two types of formula and SRR, we considered the following four subgroups: the traditional ICISS with traditional SRRs, traditional ICISS with independent SRRs, SWI with traditional SRRs and SWI with independent SWI.

### Search strategies

We defined a relevant article as any research article that reported an outcome predictive performance of any of the target tools and that was published between 1990 and 2008. We considered mortality to be an outcome for this study. We set the starting year at 1990 because the AIS currently in use was launched in 1990. We excluded articles that investigated specific age cohorts (e.g., elderly populations) and those that were limited to patients with a specific anatomical injury (e.g., head trauma patients). We also excluded studies that used the AIS 85 (or earlier versions) for score calculation.

In this systematic review, we selected the area under the Receiver Operating Characteristic curve (AUROC) as a measure of predictive performance. The AUROC is equivalent to the probability that a randomly selected subject who experienced a given event has a higher predicted risk than a randomly selected person who did not experience the event [26]. Thus, a tool with a large AUROC can accurately select patients with specific injury severities and can, in turn, reduce the selection bias for a missing target cohort. The highest AUROC is 1.0, meaning that the tool can discriminate events and non-events completely. The lowest AUROC is 0.5, meaning that the tool predicts events just by chance.

We conducted an online database search in June 2009 using MEDLINE and Embase with predetermined search words (Additional file 1). We set no language restrictions and sought a translation if required. We checked the references of relevant articles. Conference abstracts, letters and unpublished studies were not included.

### Finding relevant articles

We carefully examined the titles and abstracts of all of the articles retrieved from the online databases. We read the entire article if the relevance was unclear. After this first screening, we carefully read the complete articles to identify additional relevant articles that fulfilled the predetermined criteria described above.

### Information extraction

We extracted information relating to methodologies, study population, injury severity scoring tools and performance scores from relevant articles [see Additional file 2, which includes the information that we sought].

### Evaluating articles

We evaluated the quality of each relevant article using a quality assessment questionnaire. Because there is no widely used quality assessment tool for this type of systematic review, we developed a questionnaire to meet the needs of our study by referring to a systematic review of diagnostic tools and outcome prediction models [27-30]. Our questionnaire contains ten questions, which include two questions that were only applicable to the TRISS or the ICISS (Additional file 3). The total number of positive answers to the eight questions that could be applied to all of the target tools was reported as the overall quality score of the study. We did not sum the weights of each question because there is no consensus on how to do so.

### Statistical analysis

We did not conduct a meta-analysis in this review because we could not control the heterogeneity among studies by employing random effect models and performing subgroup analyses.

A protocol did not exist for this systematic review, and this review was not prospectively registered.

## Results

### Relevant articles

**Figure 1:**
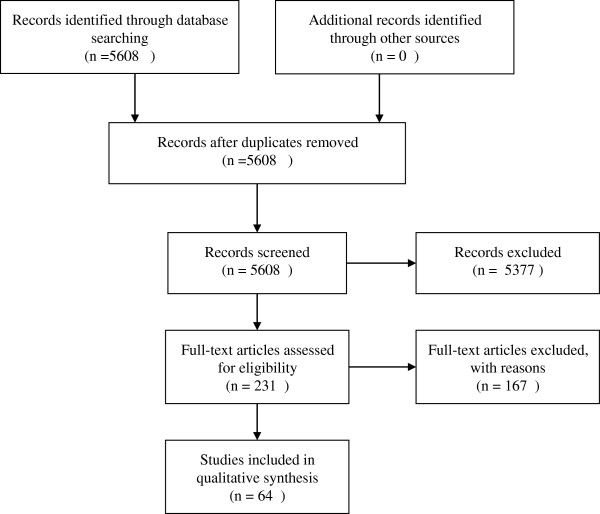
**Flow diagram of study selection process.** In total, we retrieved 5,608 potential articles from the online database search. We finally identified 64 relevant articles.

In total, we retrieved 5,608 potential articles from the online database search. After carefully reading all of the titles and abstracts and checking references using relevant journals, we finally identified 61 relevant articles written in English [3,7-17,19,31-78] and three non-English articles [79-81]. The total number of relevant articles was 64, with 157 AUROCs of the target tools (Figure 1, Table 1).

**Table 1:** List of included studies and reported AUROCs

**Author**	**Year**	**Country**	**Hospital**	**Tool**	**AUROC**	**N**	**Mortality**	**Mechanism**	**Code**	**Age**
Aydin [79]	2008	Turkey	1	ISS	0.907	550	21.6%	ND	AIS90	>16
				NISS	0.914					
				TRISS (M)	0.934					
Barbieri [31]	2004	Italy	1	TRISS (M)	0.946	93	28.0%	ND	ND	ND
Becalick [32]	2001	UK	1	TRISS (N)	0.941	677	16.2%	B + P	ND	ND
Bergeron [33]	2004	Canada	1	TRISS (M)	0.873	5,672	6.9%	B	ND	≥15
				TRISS (N)	0.878					
Bijsma [34]	2004	Netherland	1	ISS	0.84	668	18.4%	ND	ND	ND
Bonaventura [80]	2001	Czekoslovakia	1	ISS	0.57	1,113	18.0%	ND	ND	ND
				TRISS (M)	0.78					
Bouamra [35]	2006	UK	106	TRISS (N)	0.937	20,895	4.4%	B	ND	adult
Bouillon [36]	1997	Germany	32	ISS	0.961	612	30.9%	ND	AIS90	all
				TRISS (M)	0.974					
Brenneman [37]	1998	Canada	1	ISS	0.799	2,328	13.0%	B	ND	all
				NISS	0.852					
Burd [19]	2008	US	Multiple	ICISS (T)	0.726	276,366^*1^	2.8%	B + P	ICD9CM	all
				SWI (T)	0.743					
				ICISS (I)	0.793					
				ICISS (T)	0.855	312,592^*2^	5.1%			
				SWI (T)	0.866					
				ICISS (I)	0.867					
Chytra [81]	1999	Czekoslovakia	1	ISS	0.89	165^*3^	17.0%	ND	ND	≥18
				TRISS (M)	0.85					
				ISS	0.76	109^*4^	38.0%			
				TRISS (M)	0.83					
Davie [38]	2008	New Zealand	ND	ICISS (T)	0.777	186,835	5.3%	ND	ICD10AM	ND
				ICISS (T)	0.851					
Dillion [39]	2005	UK	ND	ISS	0.832	53,286	4.1%	B	ND	≥16
				NISS	0.827					
				TRISS (N)	0.939	12,606	4.4%			
DiRusso [40]	2000	US	25	ISS	0.766	2,768	8.4%	all	ND	all
				TRISS (M)	0.895^*5^					
				TRISS (M)	0.918^*6^	2,673	8.3%			
				TRISS (N)	0.92	2,768	8.4%			
Eftekhar [41]	2005	Iran	6	ISS	0.944	7,226	3.8%	all	AIS90	all
				TRISS (M)	0.969					
Fischler [42]	2007	Switzerland	1	ISS	0.75	208	13.0%	ND	ND	adult
Frankema [43]	2002	Netherland	1	TRISS (M)	0.975	1,024	6.9%	B + P	AIS90	≥15
Frankema [44]	2005	Netherland	1	TRISS (M)	0.94	1,102	11.0%	B + P	AIS90	≥15
Gabbe [45]	2005	Australia	Multiple	TRISS (M)	0.87	1,387	4.4%	B	NR	all
Glance [47]	2009	US	359	ISS	0.868	66,214	4.2%	B + P	ND	≥1
	2009	US	68	SWI (T)	0.862	749,374	5.0%	B + P	ICD9CM	≥1
Glance [46]				ICISS (T)	0.85					
Guzzo [48]	2005	US	1	ISS	0.791	2,412	15.1%	all	ND	all
				TRISS (M)	0.97					
Hannan [50]	1999	US	192	TRISS (M)	0.858	20,883	7.2%	B	AIS90	≥13
				TRISS (N)	0.857					
Hannan [7]	2005	US	192	ISS	0.776	39,534	6.9%	B	AIS90	ND
				NISS	0.786					
				ICISS (T)	0.809					
Hannan [49]	2007	US	ND	SWI (I)	0.754	117,630	2.9%	all	ICD9CM	≥12
				SWI (T)	0.764					
				ICISS (I)	0.744					
				ICISS (T)	0.745					
Harwood [51]	2006	4 countries (Germany, Netherlands, Switzerland, Austria)	82	ISS	0.78	10,062	NR	B	ND	≥16 & ≤70
				NISS	0.785					
				ISS	0.787	549	NR	P		
				NISS	0.793					
Hunter [52]	2000	UK	ND	TRISS (M)	0.9411	7,831	NR	ND	ND	ND
				TRISS (N)	0.9426					
Jamulitrat [53]	2001	Thailand	1	ISS	0.966	2,044	5.6%	all	AIS90	all
				NISS	0.974					
Kilgo [54]	2006	US	125	TRISS (M)	0.939	310,958	5.0%	B + P	ND	all
				TRISS (N)	0.95					
Kim [8]	1999	Korea	2	ISS	0.892	367	21.3%	B	ND for AIS	ND
				ICISS (T)^*7^	0.843				ICD10	
				ICISS (T)^*8^	0.909				ICD9CM	
				TRISS (N)	0.958					
Kroezen [55]	2007	Netherland	2	TRISS (M)	0.806	349	14.0%	B + P	ND	all
				TRISS (N)	0.891	179	22.0%			
Kuhls [56]	2002	US	1	ISS	0.93	3,855	3.5%	B + P	ND	all
				TRISS (M)	0.96					
Lane [57]	1996	Canada	12	TRISS (N)	0.908	1,793	7.9%	ND	AIS90	ND
Lavoie [58]	2004	Canada	3	ISS	0.818	23,306	6.3%	B	ND	≥16
				NISS	0.824					
				ISS	0.84	957	15.9%	P		
				NISS	0.824					
				ISS	0.819	24,263	6.6%	B + P		
				NISS	0.827					
Macleod [59]	2003	Uganda	1	ISS	0.811	150	25.5%	all	ND	≥16
				TRISS (M)	0.871					
Meredith [9]	2002	US	ND	ISS	0.876	76,871	5.3%	B + P	ND for AIS	all
				NISS	0.871					
				ICISS (T)	0.893				ICD9CM	
Meredith [60]	2003	US	88	ICISS (T)^*9^	0.89	170,853	5.4%	B + P	ICD9CM	all
				ICISS (T)^*10^	0.882					
Meredith [61]	2003	US	ND	ICISS (I)	0.892	192,347	5.1%	B + P	ICD9CM	all
				ICISS (T)	0.875					
Millham [62]	2004	US	ND	TRISS (M)	0.837	31,000	4.6%	B	ND	all
				TRISS (N)	0.936					
				TRISS (M)	0.982	5,200	9.9%	P		
				TRISS (N)	0.981					
Moore [10]	2008	Canada	ND	ISS	0.822	25,111	7.3%	B + P	AIS90	≥16
				NISS	0.831					
				ICISS (T)	0.852				ICD9	
Moore [63]	2009	Canada, US	Multiple	TRISS (N)	0.928	178,377	6.2%	B	ND	>16
Osler [11]	1996	US	1	ISS	0.866	2,337	NR	B	ICD9	all
				ICISS (T)	0.918					
				ISS	0.906	805	NR	P		
				ICISS (T)	0.93					
				ISS	0.87	3,142	9.0%	B + P		
				ICISS (T)	0.921					
Osler [3]	1997	US	2	ISS	0.869	3,136^*11^	9.0%	B + P	AIS90	all
				NISS	0.896					
				ISS	0.896	3,449^*12^	7.0%			
				NISS	0.907					
Osler [12]	1997	US	1	ISS	0.843	1,812	2.5%	all	ND for AIS	all
				ICISS (T)^*13^	0.884				ICD9	
				ICISS (T)^*14^	0.872					
Osler [64]	2008	US	206	ISS	0.871	140,000	4.1%	all	ND	≥1
Rabbani [65]	2007	Iran	3	TRISS (N)	0.93	2,514	6.0%	all	ND	all
Raum [66]	2009	4 countries (Germany, Netherlands, Switzerland, Austria)	97	ISS	0.722	1,292	18.9%	all	ND	≥16
				NISS	0.764					
				TRISS (M)	0.851					
									
Reiter [67]	2004	Australia	Multiple	TRISS (M)	0.84	5,538	12.3%	all	ND	≥18
Rhee [68]	1990	US	6	ISS	0.7967	691	15.8%	all	ND	≥11
Rutledge [69]	1997	US	ND	ISS	0.939	44,032	6.5%	all	ND for AIS	all
				ICISS (T)^*15^	0.939				ICD9CM	
				ICISS (T)^*16^	0.929					
				ICISS (T)^*9^	0.858					
				ICISS (T)^*17^	0.957					
Rutledge [70]	1998	US	Multiple	ICISS (T)	0.957	9,438	5.1%	all	ICD9CM	all
Rutledge [13]	1998	US	13	ISS	0.667	7,276	3.8%	all	ND for AIS	all
				ICISS (T)	0.916				ICD9CM	
				TRISS (M)	0.877					
Sacco [14]	1999	US	26	ISS	0.86	30,287	7.1%	all	ND for AIS	all
				NISS	0.86					
				ICISS (T)^*15^	0.87				ICD9CM	
				ICISS (T)^*18^	0.88					
Sammour [71]	2009	New Zealand	1	ISS	0.8547	1,197	3.7%	all	ND	≥15
				TRISS	0.963					
Stephensen [15]	2002	New Zealand	ND	ISS	0.847	340,000	1.1%	all	AIS90	all
				NISS	0.829					
				ICISS (T)	0.901				ICD9	
Suarez-Alvarez [73]	1995	Spain	1	TRISS (M)	0.85	404	19.6%	B + P	ND	ND
Tamin [72]	2008	Lebanon	1	ISS	0.881	891	3.6%	all	ND	all
				NISS	0.887					
Tay [74]	2004	US	1	ISS	0.922	NR	NR	B	ND	all
				NISS	0.923					
				ISS	0.943	NR	NR	P		
				NISS	0.924					
				ISS	0.942	6,089		B + P		
				NISS	0.936					
Ulvik [75]	2007	Norway	1	ISS	0.61	325	16.9%	ND	AIS98	>18
Vassar [76]	1999	US	6	TRISS (M)	0.82	2,414	12.3%	B + P	AIS90	≥16
West [16]	2000	US	1	ICISS (T)	0.94	9,923	NR	B + P	ICD9CM,	all
				TRISS (M)	0.947				AIS90	
Wong [77]	1996	Canada	1	TRISS (M)	0.89	470	13.0%	all	ND	all
Wong [17]	2008	Hong Kong	1	ISS	0.8677	1,166	13.8%	B	ND for AIS,	all
				ICISS (I)	0.8379				ICD9	
				ICISS (T)	0.851					
Zhao [78]	2008	China	1	ISS	0.922	1,532	NR	B	ND	≥16
				NISS	0.923					
				ISS	0.943	578	NR	P		
				NISS	0.922					
				ISS	0.943	2,110	NR	B + P		
				NISS	0.938					

AIS, Abbreviated Injury Scale; ISS, Injury Severity Score; NISS, New ISS; ICISS, International Classifications of Diseases-based (ICD-based) ISS; TRISS, Trauma and ISS: TRISS(M), TRISS with coefficients based on the Major Trauma Outcome Study (MTOS); TRISS(N), TRISS with coefficients based on non-MTOS population; ICISS–(T), product of traditional Survival Risk Ratios (SRRs); ICISS(I), product of independent SRRs; SWI, Single Worst Injury; SWI (T), SWI of traditional SRRs; SWI(I), SWI of independent SRRs.

*1, Nationwide Inpatient Sample; *2, National Trauma Data Bank (NTDB); *3, polytrauma without brain injury; *4, polytrauma with brain injury; *5, data in 1995; *6, data in 1996.

*7, SRRs derived from ICD10; *8, SRRs derived from ICD9CM; *9, SRRs derived from North Carolina Hospital Discharge Database; *10, SRRs derived from NTDB.

*11, Albuquerque data; *12, Portland data; *13, SRRs derived from Hospital Information System; *14, SRRs derived from trauma registry; *15, SRRs derived from North Carolina Trauma Registry; *16, SRRs derived from Agency for Health Care Policy Healthcare Cost Utilization Project; *17, SRRs derived from San Diego Trauma Registry; *18, SRRs derived from Pennsylvania Trauma Outcome Study.

UK, United Kingdom; US, United States of America; NZ, New Zealand.

B, blunt injury; P, penetrating injury; B + P, blunt and penetrating injuries; ND, not described; NR, not reported; NA, not applicable.

In these articles, the ISS was the most frequently studied target tool (58%), followed by the TRISS (53%), the ICISS (31%) and the NISS (25%). The MTOS TRISS were more frequently reported than the non-MTOS TRISS (see Additional file 2 for the details). Regarding the formulas used in ICISS calculation, 32 out of 39 AUROCs were derived from the traditional ICISS, whereas 7 AUROCs were derived from the SWI. There were 33 and 6 AUROCs of the ICISS using traditional and independent SRRs, respectively (Table 1).

Of the 64 relevant studies, 26 studies were conducted in the U.S., and 26 studies used data from a single hospital. Only three studies included data from hospitals in multiple countries (see Additional file 2).

### Quality assessment

**Figure 2:**
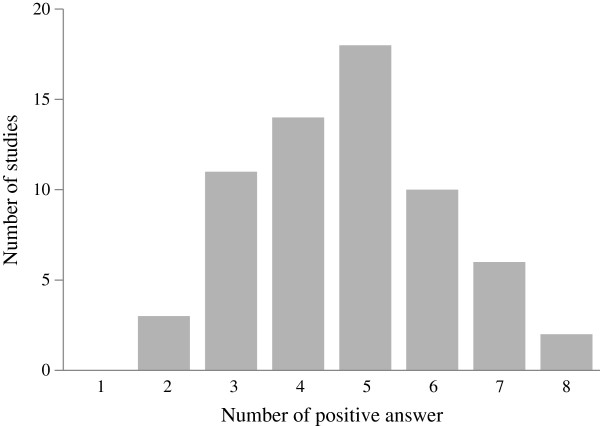
The distribution of the number of positive answers in the quality assessment questionnaire.

The results of the quality assessment are shown in Table 2. The distribution of the number of positive answers was positively skewed, and the median was 5 out of 8, ranging from 2 (4 studies) to 8 (2 studies) (Figure 2).

**Table 2:** Results of quality assessment

			
Internal validity			
Q1	Were selection criteria clearly described?		
	Yes	61	95.3%
	No	3	4.7%
Q2	Were any quality assurance measures for managing and/or collecting data described?		
	Yes	24	37.5%
	No	40	62.5%
Q3	Were missing data adequately managed?		
	Yes	38	59.4%
	No	28	43.8%
	Two studies were double-counted because a part of variable were excluded and the rest of variables were estimated.		
Q4	Was the length of follow-up described?		
	Yes	35	54.7%
	No	29	45.3%
Q5	Was the version of the reference code systems used described?		
	Yes	24	37.5%
	No	40	62.5%
Q6	Was the derivation of coefficients of TRISS or weights of ICISS described?		
	Yes	41	34.5%
	No	11	9.2%
	NA	14	11.8%
	Two studies described the derivation of only a part of scores studied.		
Q7	Were the new coefficients or weights validated?		
	Yes	25	89.3%
	No	3	10.7%
External validity			
Q8	Was the description of the study population reported?		
	Yes	62	96.9%
	No	2	3.1%
Q9	Was the study conducted using multi-institutional population?		
	Yes	28	51.9%
	No	36	48.1%
Q10	Was the precision of AUROC, such as standard error, reported?		
	Yes	31	48.4%
	No	33	51.6%

NA, not applicable; AUROC, area under the Receiver Operating Characteristic curve; TRISS, Trauma and Injury Severity Score; ICISS, International Classification of Diseases-based.

Most studies described the selection criteria for the study subjects and the demographics of the subjects. In contrast, less than half of the studies reported the following items: the version of AIS and/or ICD used (37.5%); the quality assurance measure for collecting and measuring scores (37.5%); and the precision of the AUROCs (48.4%).

Regarding the two questions that were only relevant to the TRISS and ICISS, the majority of studies reported the origin of the coefficients of the TRISS or SRRs of the ICISS (41 out of 52 studies). The TRISS and ICISS that used newly derived coefficients or SRRs were internally or externally validated in 25 out of 28 studies.

## Discussion

We identified 64 relevant articles with 157 AUROCs. The ISS was most frequently reported (48 AUROCs), followed by the TRISS (45 AUROCs), ICISS (40 AUROCs) and NISS (24 AUROCs). We could not pool the AUROCs because of the heterogeneity among the studies.

### Study quality

There was a scarcity of high-quality studies that investigated the performance of the target tools. Specifically, the version of the injury code system and any quality assurance measure were poorly described.

Most studies described their selection criteria and reported the demographic data of the study population; however, key information that can influence the predictive performance was underreported. An AUROC can be affected by two types of factors: factors that influence the measurement of injury severity scores and those that affect the outcome [82]. The former include the version of the injury codes, type of formula and derivation of coefficients and/or SRRs; the latter includes the distribution of age, mechanism of injury and inclusion/exclusion of special cohorts (e.g., elderly patients with an isolated hip fracture, dead on hospital arrival). One of these factors (the version of the injury code system) was found to be underreported by the questionnaire. These factors may need to be fully described as much as possible to improve the quality of studies on injury severity scoring tools.

### Source of heterogeneity

The sources of the heterogeneity among the relevant studies could be found in the different characteristics of their study populations. For instance, we found a wide range of crude mortality rates of the study populations, ranging from 1.1% [15] to 38% [81]. This wide distribution of the rates might be due to the difference in the type of database used between the ICISS and AIS-based tools. Studies that investigated the ICISS mostly used administrative databases, whereas studies that analyzed AIS-based tools generally used a trauma registry. Because the majority of studies of the ICISS used such a database without considering the mechanism of injury, severity of injury or, sometimes, age groups, the crude mortality rates of these studies were lower than those of the studies of AIS-based tools. Among 19 studies of the ICISS, only two studies reported that their crude mortality rates were more than 10%, whereas the rates of all of the other studies were less than 10%. In contrast, among 45 articles that did not study the ICISS, 22 studies reported more than 10% as the crude mortality rate. These high mortality rates of studies of the AIS-based tools may be explained by the fact that these studies used trauma registries that generally have inclusion and/or exclusion criteria that prevent many minor injuries from being registered.

### ISS vs. NISS

We identified 16 studies that reported 24 pairs of AUROCs of the ISS and NISS [3,7,9,10,14,15,37,39,51,53,58,66,72,74,78,79]. Among the 24 pairs of AUROCs, eight pairs demonstrated that the ISS had a greater AUROC than the NISS [9,15,39,58,74,78], whereas the other 16 pairs showed greater AUROCs for the NISS than for the ISS. There were seven pairs of AUROCs that were derived using only blunt trauma patients [7,37,39,51,58,74,78]. Among these seven pairs, only one pair had a higher AUROC for the ISS than the NISS [39]. There were four pairs of AUROCs that were derived using penetrating trauma patients [51,58,74,78]. Only one study had a higher AUROC for the NISS than the ISS [51]. Although further studies are required, the NISS might be better at predicting the outcomes of blunt trauma patients than the ISS, and vice versa for penetrating trauma patients. Because the mechanism of injury might affect the predictive performance of the ISS and the NISS, researchers should clearly describe the mechanism of injury of the study population and analyze blunt and penetrating trauma patients separately when investigating the predictive performance.

### ICISS vs. AIS-based tools

We could not clearly determine the relative performance of the ICISS against the AIS-based tools because of the scarcity of comparative studies. We identified 11 studies that reported AUROCs of the ICISS and ISS and/or NISS [8-15,17,49,69]. Most of these studies reported greater AUROCs for the ICISS than for the ISS/NISS, with one exception [17]. In contrast, the ICISS was rarely compared with the TRISS. We could find three studies that reported AUROCs of both the ICISS and the TRISS [8,13,16]. Among these studies, two studies showed that the TRISS performed better than the ICISS [8,13], and one study demonstrated the opposite [16]. Based on these results, the ICISS is better at predicting outcomes than the ISS/NISS, but the superiority of the TRISS over the ICISS was inconclusive.

### Instability of the ICISS

The ICISS appeared to be unstable in terms of its predictive performance for two reasons: the multiplicity of its formula and SRR and the dependency on the data source for the SRRs. We divided the AUROCs of the ICISS into four subgroups based on the formula and type of SRR. There was only one study that reported AUROCs of all four types of the ICISS [49]. According to this study, the SWI with traditional SRRs performed best (AUROC = 0.764), followed by the SWI with independent SRRs (0.754), the traditional ICISS with traditional SRRs (0.745) and the traditional ICISS with independent SRRs (0.744). Glance et al. supported the superiority of the SWI over the traditional ICISS [47], but Burd et al. reported a greater AUROC for the traditional ICISS than for the SWI [19]. Regarding the type of SRRs, there were three studies that compared the traditional SRRs with independent SRRs [17,49,61]. The results were inconclusive; one reported that independent SRRs were better than traditional SRRs, but the other two reported the opposite.

The predictive performance of the ICISS was also dependent on the data sources from which the SRRs were derived. Rutledge et al. reported AUROCs of traditional ICISSs using different sets of SRRs derived from four different databases [69]. One of the four AUROCs was greater than that of the ISS, but the other three AUROCs were the same as or less than that of the ISS. Kim et al. demonstrated another type of difference in the source data of SRRs. These authors showed that the traditional ICISS based on the ICD9CM performed better than the ISS but that the traditional ICISS using the ICD10 performed worse than the ISS. As a whole, the type of data used for SRR derivation appeared to be a crucial factor in determining the predictive performance of the ICISS.

### Generalizability

It is difficult to draw broad generalizations from this study because 41% of the studies evaluated were conducted in the U.S., and 41% of the studies contained data from a single hospital (see Additional file 2 for the details). In short, the results derived from narrowly recruited study populations cannot be readily applied to other populations. One can increase the generalizability of results with data from multiple hospitals and/or multiple countries. Trauma registries in which multiple countries take part have recently been developed [83,84]. The use of such registries might constitute an alternative way to increase the generalizability of study results.

### Potential biases

We searched relevant articles using two major online databases, MEDLINE and Embase. We set no language restrictions and checked the references of the relevant articles. These processes enabled us to identify as many relevant articles as possible and to reduce dissemination bias. We might have been able to reduce the bias further if we used other databases (e.g., CINAHL), although the effect of adding another database might have been minimal.

### Limitations

We only focused on four injury severity scoring systems. We acknowledge that there are other tools, including A Severity Characterization of Trauma (ASCOT) [85], the Anatomic Profile Score (APS) and the modified Anatomic Profile (mAP) [86]. However, because these tools were not widely used when this study was conducted, we excluded these tools from this review.

### Future research directions

Future studies might need to focus more on statistical models that incorporate an injury severity scoring tool with a risk adjustment. Such models could potentially yield a higher predictive performance than the tools in this review. Moore et al. reported on the Trauma Risk Adjustment Model (TRAM), which was superior to the TRISS with regard to both discrimination and calibration [63]. Such high performance predictive models play a key role in hospital performance rankings (e.g., the Trauma Quality Improvement Program) [87]. Furthermore, although systematic reviews studying predictive models for brain trauma injury have been conducted [28], a review that focuses on predictive models for general trauma populations, including the TRAM, has not yet been performed. Reviewing the statistical models used to predict the outcomes of injured patients would provide researchers with clues for important predictors and appropriate statistical techniques.

## Conclusions

We could not pool reported evidence because of the heterogeneity among the relevant studies. The NISS appeared to be better at predicting the mortality of blunt trauma patients than the ISS. We could not determine the relative performance of the ICISS against the TRISS. The ICISS appeared to be less stable in its predictive performance than the AIS-based tools because of the many variations in its computational method. Additional studies are required to standardize the derivation of the ICISS and determine the relative performance of the ICISS against the AIS-based tools.

## Competing interests

The authors declare that they have no competing interests.

## Authors’ contributions

HT and IJ designed the study and directed its implementation. DM, NG and AY helped to design the statistical strategy of the study. IJ helped to extract data and assess the quality of the studies. HT analyzed the data and wrote the paper. All authors read and approved the final manuscript.

## Supplementary Material

Additional file 1 MEDLINE and Embase search strategy using OvidSP version 3. Click here for file

Additional file 2 Study characteristics of the relevant studies. Details of study characteristics. Click here for file

Additional file 3 Quality assessment questionnaire of injury severity scoring tools. Click here for file
