# Supplementary material for: Systematic review of predictive performance of injury severity scoring tools
Source: Scand J Trauma Resusc Emerg Med. 2012 Sep 10;20:63. doi: 10.1186/1757-7241-20-63 (PMC3511252; doi:10.1186/1757-7241-20-63)
Supplement: Additional file 1 — MEDLINE and Embase search strategy using OvidSP version 3. [file 1757-7241-20-63-S1.ppt]

## Slide 1
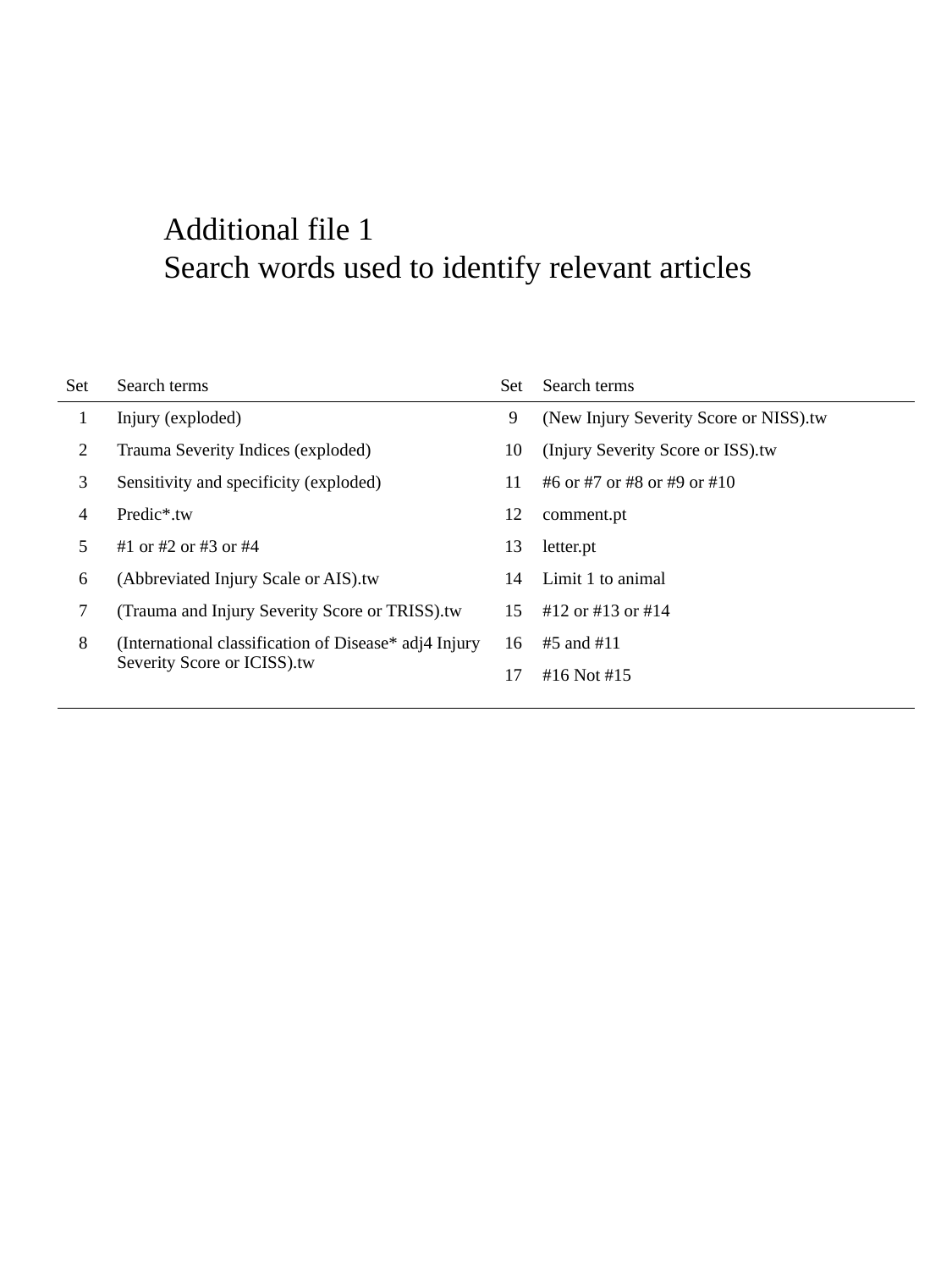

Additional file 1
Search words used to identify relevant articles
| | | | |
| --- | --- | --- | --- |
| Set | Search terms | Set | Search terms |
| 1 | Injury (exploded) | 9 | (New Injury Severity Score or NISS).tw |
| 2 | Trauma Severity Indices (exploded) | 10 | (Injury Severity Score or ISS).tw |
| 3 | Sensitivity and specificity (exploded) | 11 | #6 or #7 or #8 or #9 or #10 |
| 4 | Predic\*.tw | 12 | comment.pt |
| 5 | #1 or #2 or #3 or #4 | 13 | letter.pt |
| 6 | (Abbreviated Injury Scale or AIS).tw | 14 | Limit 1 to animal |
| 7 | (Trauma and Injury Severity Score or TRISS).tw | 15 | #12 or #13 or #14 |
| 8 | (International classification of Disease\* adj4 Injury Severity Score or ICISS).tw | 16 | #5 and #11 |
| | | 17 | #16 Not #15 |
