# Supplementary material for: Systematic review of predictive performance of injury severity scoring tools
Source: Scand J Trauma Resusc Emerg Med. 2012 Sep 10;20:63. doi: 10.1186/1757-7241-20-63 (PMC3511252; doi:10.1186/1757-7241-20-63)
Supplement: Additional file 2 — Study characteristics of the relevant studies. Details of study characteristics. [file 1757-7241-20-63-S2.doc]

| Study characteristics of relevant studies (Total=64) | | | | | | | | | | | | | | | |  |  |  | |  |
| --- | --- | --- | --- | --- | --- | --- | --- | --- | --- | --- | --- | --- | --- | --- | --- | --- | --- | --- | --- | --- |
|  |  |  |  |  |  |  |  |  |  |  |  |  |  |  | n |  | (%) |  | |  |
| Injury severity scoring tools investigated | | | | | | | | | | | | |  |  |  |  |  |  | |  |
|  | ISS | | |  |  |  |  |  |  |  |  |  |  |  | 37 |  | (58) |  | |  |
|  | TRISS | | |  |  |  |  |  |  |  |  |  |  |  | 34 |  | (53) |  | |  |
|  | ICISS | |  |  |  |  |  |  |  |  |  |  |  |  | 19 |  | (30) |  | |  |
|  | NISS | | |  |  |  |  |  |  |  |  |  |  |  | 16 |  | (25) |  | |  |
|  |  |  |  |  |  |  |  |  |  |  |  |  |  |  |  |  |  |  | |  |
| Year of publication | | | | | | |  |  |  |  |  |  |  |  |  |  |  |  | |  |
|  | 1990-1995 | | | |  |  |  |  |  |  |  |  |  |  | 2 |  | (3) |  | |  |
|  | 1996-2000 | | | |  |  |  |  |  |  |  |  |  |  | 18 |  | (28) |  | |  |
|  | 2001-2005 | | | |  |  |  |  |  |  |  |  |  |  | 23 |  | (36) |  | |  |
|  | 2006-2009 | | | |  |  |  |  |  |  |  |  |  |  | 21 |  | (33) |  | |  |
|  |  |  |  |  |  |  |  |  |  |  |  |  |  |  |  |  |  |  | |  |
| Country of study | | | | | |  |  |  |  |  |  |  |  |  |  |  |  |  | |  |
|  | US | |  |  |  |  |  |  |  |  |  |  |  |  | 26 |  | (41) |  |  | |
|  | Canada | | |  |  |  |  |  |  |  |  |  |  |  | 6 |  | (9) |  |  | |
|  | UK | |  |  |  |  |  |  |  |  |  |  |  |  | 4 |  | (6) |  |  | |
|  | Netherland | | | |  |  |  |  |  |  |  |  |  |  | 4 |  | (6) |  |  | |
|  | New Zealand | | | | |  |  |  |  |  |  |  |  |  | 3 |  | (5) |  |  | |
|  | Australia, China, Czechoslovakia, Iran | | | | | | | | | | | | |  | 2 each |  | (13) |  |  | |
|  | Germany, Italy, Korea, Lebanon, | | | | | | | | | | | |  |  | 1 each |  | (16) |  |  | |
|  | Norway, Spain, Switzerland, Thailand | | | | | | | | | | | | |  |  |  |  |  | |  |
|  | Turkey, Uganda | | | | | |  |  |  |  |  |  |  |  |  |  |  |  | |  |
|  | Multi-country study | | | | | | | | | | | |  |  | 3 |  | (5) |  |  | |
|  |  |  |  |  |  |  |  |  |  |  |  |  |  |  |  |  |  |  | |  |
| Data collection | | | | |  |  |  |  |  |  |  |  |  |  |  |  |  |  | |  |
|  | Extracted from existing database | | | | | | | | | | |  |  |  | 42 |  | (65) |  | |  |
|  | Prospectively collected | | | | | | | |  |  |  |  |  |  | 16 |  | (25) |  | |  |
|  | Retrospective chart review | | | | | | | | |  |  |  |  |  | 3 |  | (5) |  | |  |
|  | ND | |  |  |  |  |  |  |  |  |  |  |  |  | 3 |  | (5) |  | |  |
|  |  |  |  |  |  |  |  |  |  |  |  |  |  |  |  |  |  |  | |  |
| Number of participating hospitals | | | | | | | | | | |  |  |  |  |  |  |  |  | |  |
|  | Single | | |  |  |  |  |  |  |  |  |  |  |  | 26 |  | (41) |  | |  |
|  | Multiple | | |  |  |  |  |  |  |  |  |  |  |  | 28 |  | (44) |  | |  |
|  | ND | |  |  |  |  |  |  |  |  |  |  |  |  | 10 |  | (16) |  | |  |
|  |  |  |  |  |  |  |  |  |  |  |  |  |  |  |  |  |  |  | |  |
| Age limit of inclusion | | | | | | |  |  |  |  |  |  |  |  |  |  |  |  | |  |
|  | All age | | |  |  |  |  |  |  |  |  |  |  |  | 29 |  | (44) |  | |  |
|  | ≧ 16 years old | | | | | |  |  |  |  |  |  |  |  | 8 |  | (13) |  | |  |
|  | ≧ 1 years old | | | | |  |  |  |  |  |  |  |  |  | 3 |  | (5) |  | |  |
|  | Other ages | | | |  |  |  |  |  |  |  |  |  |  | 14 |  | (22) |  | |  |
|  | ND | |  |  |  |  |  |  |  |  |  |  |  |  | 10 |  | (16) |  | |  |
|  |  |  |  |  |  |  |  |  |  |  |  |  |  |  |  |  |  |  | |  |
| Mechanism of injury | | | | | | |  |  |  |  |  |  |  |  |  |  |  |  | |  |
|  | blunt and penetrationg injury | | | | | | | | | |  |  |  |  | 23 |  | (36) |  | |  |
|  | all types of injury | | | | | |  |  |  |  |  |  |  |  | 20 |  | (31) |  | |  |
|  | blunt injury | | | |  |  |  |  |  |  |  |  |  |  | 10 |  | (16) |  | |  |
|  | N D | |  |  |  |  |  |  |  |  |  |  |  |  | 11 |  | (17) |  | |  |
|  |  |  |  |  |  |  |  |  |  |  |  |  |  |  |  |  |  |  | |  |
| Proportion of blunt injuries | | | | | | | | |  |  |  |  |  |  |  |  |  |  | |  |
|  | 100% | | |  |  |  |  |  |  |  |  |  |  |  | 10 |  | (16) |  | |  |
|  | 90-99% | | |  |  |  |  |  |  |  |  |  |  |  | 7 |  | (11) |  | |  |
|  | 80-89% | | |  |  |  |  |  |  |  |  |  |  |  | 8 |  | (13) |  | |  |
|  | 70-79% | | |  |  |  |  |  |  |  |  |  |  |  | 1 |  | (2) |  | |  |
|  | 60-69% | | |  |  |  |  |  |  |  |  |  |  |  | 1 |  | (2) |  | |  |
|  | multiple datasets with different %blunt | | | | | | | | | | | | |  | 3 |  | (5) |  | |  |
|  | NR | |  |  |  |  |  |  |  |  |  |  |  |  | 34 |  | (51) |  | |  |
|  |  |  |  |  |  |  |  |  |  |  |  |  |  |  |  |  |  |  | |  |
| Pre-hospital death | | | | | | |  |  |  |  |  |  |  |  |  |  |  |  | |  |
|  | Included | | |  |  |  |  |  |  |  |  |  |  |  | 1 |  | (2) | | |  |
|  | Excluded | | | |  |  |  |  |  |  |  |  |  |  | 24 |  | (38) |  | |  |
|  | ND | |  |  |  |  |  |  |  |  |  |  |  |  | 39 |  | (60) |  | |  |

|  |  |  |  |  |  |  |  |  |  |  |  |  |  |  |  |  |  |  |  |
| --- | --- | --- | --- | --- | --- | --- | --- | --- | --- | --- | --- | --- | --- | --- | --- | --- | --- | --- | --- |
| Isolated hip fracture | | | | | | |  |  |  |  |  |  |  |  |  |  |  |  |  |
|  | Included | | |  |  |  |  |  |  |  |  |  |  |  | 0 |  | (0) | |  |
|  | Excluded | | | |  |  |  |  |  |  |  |  |  |  | 7 |  | (11) |  |  |
|  | ND | |  |  |  |  |  |  |  |  |  |  |  |  | 57 |  | (89) |  |  |
|  |  |  |  |  |  |  |  |  |  |  |  |  |  |  |  |  |  |  |  |
| Validation | | | |  |  |  |  |  |  |  |  |  |  |  |  |  |  |  |  |
|  |  | split sample | | | |  |  |  |  |  |  |  |  |  | 7 |  | (11) |  |  |
|  |  | external | | |  |  |  |  |  |  |  |  |  |  | 10 |  | (16) |  |  |
|  |  | bootstrapping | | | | |  |  |  |  |  |  |  |  | 5 |  | (8) |  |  |
|  |  | cross-validation | | | | | |  |  |  |  |  |  |  | 3 |  | (5) |  |  |
|  |  | Not validated | | | | |  |  |  |  |  |  |  |  | 3 |  | (5) |  |  |
|  |  | NA | |  |  |  |  |  |  |  |  |  |  |  | 36 |  | (56) |  |  |
|  |  |  |  |  |  |  |  |  |  |  |  |  |  |  |  |  |  |  |  |
| NA: not assessed; ND: not described; NR: not reported; SRR: survival risk ratio | | | | | | | | | | | | | | | | | | | |
| MTOS: Major Trauma Outcome Study | | | | | | | | | | | | |  |  |  |  |  |  |  |
