# Supplementary material for: Systematic review of predictive performance of injury severity scoring tools
Source: Scand J Trauma Resusc Emerg Med. 2012 Sep 10;20:63. doi: 10.1186/1757-7241-20-63 (PMC3511252; doi:10.1186/1757-7241-20-63)
Supplement: Additional file 3 — Quality assessment questionnaire of injury severity scoring tools. [file 1757-7241-20-63-S3.ppt]

## Slide 1
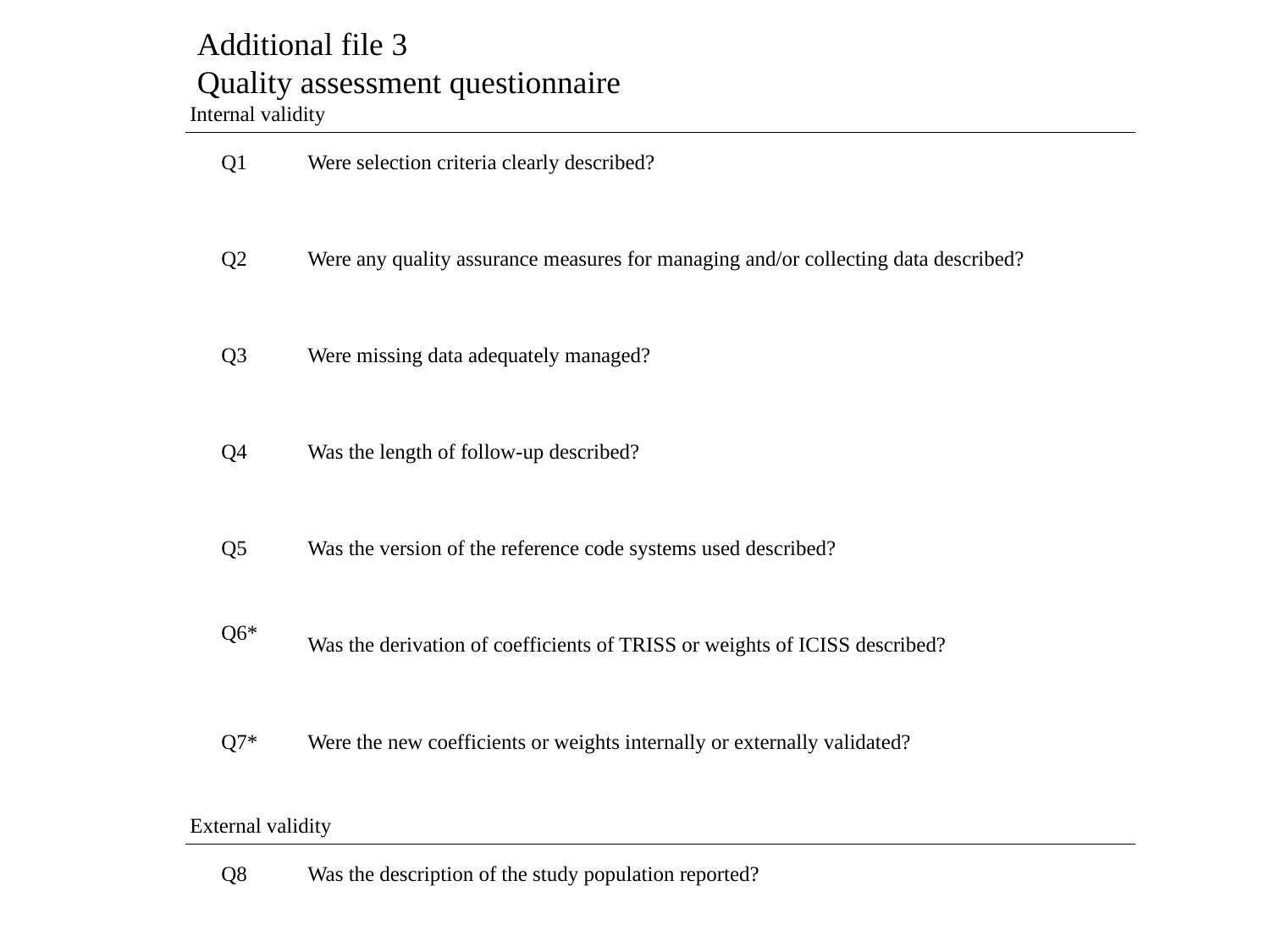

Additional file 3
Quality assessment questionnaire
| Internal validity | | | | | | | | | |
| --- | --- | --- | --- | --- | --- | --- | --- | --- | --- |
| | Q1 | Were selection criteria clearly described? | | | | | | | |
| | | | | | | | | | |
| | Q2 | Were any quality assurance measures for managing and/or collecting data described? | | | | | | | |
| | | | | | | | | | |
| | Q3 | Were missing data adequately managed? | | | | | | | |
| | | | | | | | | | |
| | Q4 | Was the length of follow-up described? | | | | | | | |
| | | | | | | | | | |
| | Q5 | Was the version of the reference code systems used described? | | | | | | | |
| | | | | | | | | | |
| | Q6\* | Was the derivation of coefficients of TRISS or weights of ICISS described? | | | | | | | |
| | | | | | | | | | |
| | Q7\* | Were the new coefficients or weights internally or externally validated? | | | | | | | |
| | | | | | | | | | |
| External validity | | | | | | | | | |
| | Q8 | Was the description of the study population reported? | | | | | | | |
| | | | | | | | | | |
| | Q9 | Was the study conducted using multi-institutional population? | | | | | | | |
| | | | | | | | | | |
| | Q10 | Was the precision of AUROC, such as standard error, reported? | | | | | | | |
| \*: applicable to the TRISS and ICISS only. | | | | | | | | | |
